# The effects of direct hemoperfusion using a polymyxin B-immobilized column in a pig model of severe *Pseudomonas aeruginosa* pneumonia

**DOI:** 10.1186/s13613-016-0155-3

**Published:** 2016-07-05

**Authors:** Gianluigi Li Bassi, Joan Daniel Marti, Eli Aguilera Xiol, Talitha Comaru, Francesca De Rosa, Montserrat Rigol, Silvia Terraneo, Mariano Rinaudo, Laia Fernandez, Miguel Ferrer, Antoni Torres

**Affiliations:** Division of Animal Experimentation, Department of Pulmonary and Critical Care Medicine, Thorax Institute, Hospital Clínic, Barcelona, Spain; Institut d’Investigacions Biomèdiques August Pi i Sunyer (IDIBAPS), Barcelona, Spain; Centro de Investigación Biomedica En Red- Enfermedades Respiratorias (CIBERES), Majorca, Spain; University of Barcelona, Barcelona, Spain; University of Milan, Milan, Italy; Research Laboratory, Department of Pulmonary and Critical Care Medicine, Hospital Clinic, Barcelona, Spain

**Keywords:** Lung—endotoxemia, Intensive care—pulmonary, Complications—septicemia, Infection—bacterial

## Abstract

**Background:**

Hemoperfusion through a column containing polymyxin B-immobilized fiber (PMX-HP) is beneficial in abdominal sepsis. We assessed the effects of PMX-HP in a model of severe *Pseudomonas aeruginosa* pneumonia.

**Methods:**

Eighteen pigs with severe *P. aeruginosa* pneumonia were mechanically ventilated for 76 h. Pigs were randomized to receive standard treatment with fluids and vasoactive drugs, or standard treatment with two 3-h PMX-HP sessions. Antibiotics against *P. aeruginosa* were never administered. We assessed endotoxemia through the endotoxin activity assay (EA). We measured the static lung elastance, ratio of arterial partial pressure per inspiratory fraction of oxygen (PaO_2_/FIO_2_), mean arterial pressure, cardiac output, systemic vascular resistance and inotropic score. Finally, every 24 h, we assessed complete blood count.

**Results:**

In comparison with the control group, PMX-HP decreased percentage of circulating neutrophils from 47.4 ± 13.8 to 40.8 ± 11.5 % (*p* = 0.009). In a subgroup of animals with the worst hemodynamic impairment, EA in the control and PMX-HP groups was 0.50 ± 0.29 and 0.29 ± 0.14, respectively (*p* = 0.018). Additionally, in the control and PMX-HP groups, static lung elastance was 26.9 ± 8.7 and 25.3 ± 7.5 cm H_2_O/L (*p* = 0.558), PaO_2_/FIO_2_ was 347.3 ± 61.9 and 356.4 ± 84.0 mmHg (*p* = 0.118), mean arterial pressure was 81.2 ± 10.3 and 81.6 ± 13.1 mmHg (*p* = 0.960), cardiac output was 3.30 ± 1.11 and 3.28 ± 1.19 L/min (*p* = 0.535), systemic vascular resistance was 1982.6 ± 608.4 and 2011.8 ± 750.0 dyne/s/cm^–5^ (*p* = 0.939), and inotropic score was 0.25 ± 0.10 and 0.26 ± 0.18 (*p* = 0.864).

**Conclusions:**

In mechanically ventilated pigs with severe *P. aeruginosa* pneumonia, PMX-HP does not have any valuable clinical benefit, and studies are warranted to fully evaluate a potential role of PMX-HP in septic shock associated with severe pulmonary infections.

**Electronic supplementary material:**

The online version of this article (doi:10.1186/s13613-016-0155-3) contains supplementary material, which is available to authorized users.

## Background

Bacterial endotoxin is a crucial component of the outer membrane of Gram-negative bacteria. During the course of an infection, the lipid A component of the molecule is released by bacteria that duplicate or die [1]. This activates a vivid inflammatory response by the host immunitary system and plays a crucial role in the development of sepsis and associated organ failure. Indeed, administration of endotoxin in healthy humans increases circulating cytokines and activates coagulation and complement pathways, mimicking the classical clinical signs of sepsis [2, 3].

In the latest decade, there has been an increasing interest in selective removal of endotoxin from blood to reduce the burden of sepsis [1, 4]. In particular, polymyxin B is an antibiotic with the attractive ability to bind and inactivate endotoxin. Yet, the systemic use of this drug is limited by the associated neurotoxicity and nephrotoxicity. In the 1990s, a novel affinity column, characterized by polymyxin B, covalently immobilized to a polystyrene-derived fiber, was developed in Japan [5]. When blood passes through this column, via an extracorporeal circuit, endotoxin is efficiently cleared, with no detectable polymyxin B in the elute [6]. In a pilot trial [7] in septic patients, hemoperfusion through the column containing polymyxin B-immobilized fiber (PMX-HP) produced a significant improvement in hemodynamic parameters. In the EUPHAS trial [8], 64 patients were treated, following abdominal surgery, with two 2-h sessions of PMX-HP. The 28-day absolute risk of death improved from 53 to 32 % (*p* = 0.03). Conversely, in a recently completed trial [9], patients who underwent surgical procedures for abdominal infections were randomized to standard or PMX-HP treatment. PMX-HP was not associated with any survival benefit, and it did not reduce risks for developing organ failures. Importantly, in both studies only patients with abdominal sepsis, who promptly underwent surgery to control the primary source of infection, were enrolled. Thus, PMX-HP was primarily aimed at clearing the residual circulating endotoxin.

Besides the effects of PMX-HP on sepsis-associated cardiovascular dysfunction, a few investigators have suggested that PMX-HP could also improve pulmonary function in exacerbated idiopathic pulmonary fibrosis [10], interstitial pneumonia [11] and acute lung injury (ALI) [12–15]. In particular, patients with pulmonary comorbidities, hospitalized for prolonged periods or undergoing invasive mechanical ventilation (MV) present the greatest risks for developing pneumonia by Gram-negative pathogens [16–18]. Yet, to date there is a lack of evidence on the potential benefits of PMX-HP in the early stages of severe Gram-negative pneumonia. Thus, we designed a prospective randomized trial in pigs with severe *Pseudomonas aeruginosa* and on MV for 76 h to examine whether PMX-HP would reduce endotoxin activity (EA) and consequently improve hemodynamic, pulmonary and clinical variables.

## Methods

The study protocol was approved by the Animal Experimentation Ethics Committee of the University of Barcelona. Animals were managed according to the Declaration of Helsinki conventions for the use and care of animals.

### Study animals, handlings, end of the study

Eighteen Large White–Landrace female pigs (weight 32.8 ± 2.9 kg) were induced [19], intubated and connected to a mechanical ventilator (SERVO-I, Maquet, NJ, USA). Anesthesia was maintained with a continuous infusion of midazolam, 0.2–0.8 mg/kg/h, and fentanyl, 5–10 µg/kg/h, in order to maintain cessation of spontaneous movements following painful stimulation. Pigs were ventilated in volume control, tidal volume 10 mL/kg, positive end-expiratory pressure (PEEP) and respiratory rate adjusted to maintain gas exchange within the physiologic range. Inspiratory gases were conditioned through a heated humidifier (Conchatherm III, Hudson RCI, Temecula, CA). Throughout the study, lactated Ringer’s and 0.9 % NaCl solutions in a 1:1 ratio were administrated at 0.5–3 mL/kg/h. Ceftriaxone was administered to prevent pulmonary endogenous colonization. The femoral artery was cannulated for systemic arterial pressure monitoring and collection of blood samples. As previously described [19], we inserted a Swan–Ganz catheter into the jugular vein to monitor pulmonary artery pressure (PAP), central venous pressure (CVP), pulmonary artery wedge pressure (PCWP), core blood temperature and cardiac output (CO). A Foley catheter was introduced into the bladder to monitor urinary output. Animals were euthanized after 76 h of invasive MV or when severe respiratory or hemodynamic instability was sustained, irrespective of maximal ventilatory (inspiratory fraction of oxygen of 1 and PEEP ≥15 cm H_2_O) or hemodynamic support (fluid challenge ≥1.5 L and norepinephrine ≥3 µg/kg/min). Upon autopsy, we took a tissue sample from each lobe for quantitative culture [20].

### Model of severe *P. aeruginosa* pneumonia

As previously described [21], after surgical preparation and stabilization, 15 mL of a 10^8^ colony-forming unit (cfu)/mL suspension of *P. aeruginosa* ATCC 27853 was inoculated through sequential insertion of a bronchoscope into the main right upper, medium and lower bronchi and the main left upper and lower bronchi. Per each bronchus, the bacterial suspension was slowly instilled over 30 s. This model is characterized by severe impairment of pulmonary function and mortality of 66 % before the end of the 72-h study [21]. Thus, to improve survival and ensure both PMX-HP treatments, we slightly modified the original ventilatory settings. In particular, in comparison with the aforementioned study, we decreased the tidal volume from 15 to 10 mL/kg and we applied PEEP. We never administered antibiotics active against *P. aeruginosa*. Blood was cultured daily.

### Randomization

After bacterial inoculation, pigs were randomized into the following groups through a computer-generated random numbers using block randomization, with a fixed block length of 18 animals:

Control group: Standard treatment to sustain hemodynamic stability, according to the surviving sepsis campaign guidelines [22], was applied. In particular, crystalloids (sequential fluid challenges of 250 mL) and norepinephrine (0.2–3 µg/kg/min) were administered to achieve a mean arterial pressure (MAP) ≥65 mm Hg; urine output ≥0.5 m/kg/h and mixed venous oxygen saturation (SvO_2_) ≥65 %.

Treatment group: During surgical preparation, an 11-Fr dual lumen catheter (Fresenius Medical Care España, S.A., Madrid, Spain) was inserted into the femoral vein. As detailed above, standard treatment was applied. Additionally, after 24 and 48 h from pulmonary bacterial challenge, PMX-HP was performed for 3 h through a veno-venous, pump-driven, extracorporeal system (EstorFLOW^®^, Estor, Milan, Italy) and a column containing polymyxin B-immobilized fiber (Toraymyxin, Toray Medical Co., Ltd, Tokyo, Japan). Additional file 1: Figure S1 depicts experimental settings during PMX-HP. Blood flow through the column was maintained between 80 and 100 mL/min. During the procedure, heparin was continuously administered and adjusted to prolong the partial thromboplastin time (PTT) 1.5–2.0 times the mean control value.

### Endotoxin activity assay

The EA assay was measured every 12 h and after each PMX-HP, as previously described [23, 24]. The assay measures EA, based on whole blood neutrophil chemiluminescence activity (on a scale from 0 to 1). EA was assayed in duplicate, and samples were considered adequate if the coefficient of variation between duplicates was lower than 30 %. As detailed in the Additional file 2: Appendix, we performed preliminary studies to estimate endotoxin concentration in pig blood, based on the EA. Thus, as predicted by the regression analysis reported in the Additional file 2: Appendix, EA level of 0 is equivalent to *P. aeruginosa* endotoxin concentration of 0 pg/mL, a level of 0.4–350.15 pg/mL and a level of 0.6–1361.36 pg/mL.

### Respiratory measurements

Every 24 h, airway and esophageal pressures and respiratory flow rates were measured and recorded [20]. The static lung elastance was computed using standard formulae [20].

### Hemodynamic measurements and gas exchanges

Every 12 h, gas exchange (arterial and mixed venous blood), MAP, CVP, mean PAP, PCWP and CO were measured. Stroke volume (SV), systemic vascular resistance (SVR), pulmonary vascular resistance (PVR), venous admixture, venous-to-arterial partial pressure carbon dioxide difference (Pv-aCO_2_) and fluid balance were computed. As previously reported [8], we computed the inotropic score and the vasopressor dependency index.

### Clinical variables

Every 24 h, we assessed complete blood count, body temperature, coagulation parameters and alanine transaminase.

### Statistical analysis

Continuous variables were described as means and standard deviations, unless otherwise specified. Categorical variables were described as frequencies and percentages. Continuous variables were analyzed using a restricted maximum likelihood analysis, based on repeated measures approach, including the independent ‘treatment’ variable, the repeated ‘time of assessment’ variable and their interaction as factors. Per each model, normality of residuals was confirmed through visual inspection of residuals distribution. A compound symmetry (co)variance structure was used to model the within-subject errors. Each pairwise comparison was corrected using Bonferroni’s test, in order to control for the experiment-wise error rate. All reported *p* values are the exact *p* values derived through a permutation test. A two-sided *p* value ≤0.05 was considered statistically significant. All statistical analyses were performed using SAS software (version 9.2; SAS Institute, Cary, NC).

## Results

Nine animals were enrolled into the control group and eight into the treatment group. One animal of the treatment group was euthanized after 9 h for surgical complications, and it was excluded from the final analysis. Additionally, one control animal was euthanized after 64 h of MV for severe respiratory instability. Finally, in one treatment animal, the study was terminated after 62 h of MV, due to accidental extubation. In the treatment and control group, lung tissue *P. aeruginosa* concentration was 3.65 ± 1.01 and 3.90 ± 1.54 log cfu/g, respectively (*p* = 0.341). *P. aeruginosa* bacteremia was found only in one control animal. Analysis of the worst clinical parameters recorded within the first 24 h of the study is reported in Table 1. No significant differences were found, corroborating similar clinical conditions before study treatments.

**Table 1:** Analysis of worst clinical parameters, within 24 h from bacterial inoculation

PIG N.	Highest heart rate within 24 h from bacterial challenge (beats/min)	Baseline mean arterial pressure (mm Hg)	Greatest mean arterial pressure drop within 24 h from bacterial challenge (%)	Highest body temperature within 24 h from bacterial challenge (°C)	Highest WBC within 24 h from bacterial challenge (µL^−1^)
Control group (9 animals)	86 ± 22	82.1 ± 7.2	11.4 ± 3.4	39.5 ± 0.9	21,544 ± 6323
Treatment group (8 animals)	95 ± 23	78.5 ± 11.2	9.7 ± 8.9	40.0 ± 0.6	16,162 ± 4785
*p* value	0.402	0.438	0.630	0.188	0.069

We report analyses of worst clinical parameters (within 24 h from bacterial challenge) to confirm the presence of pneumonia and investigate potential baseline differences between study groups. Data are reported as mean ± standard deviation

*MV* mechanical ventilation, *WBC* white blood cell

### Hemodynamics

**Fig. 1:**
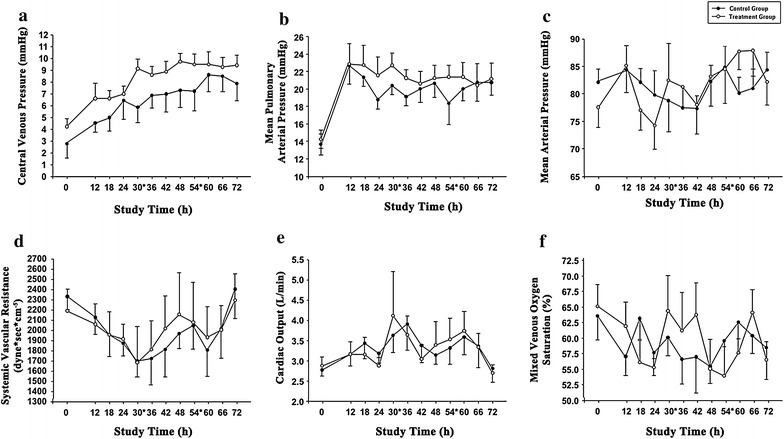
Differences in most significant hemodynamic parameters, between the control and treatment group. **a** Central venous pressure significantly differed between groups (*p* < 0.001) and changed over time (*p* < 0.001), without interactions between study groups and time of assessment. Similarly, pulmonary arterial pressure (**b**) significantly differed between groups (*p* = 0.006) and significantly changed over time (*p* < 0.001). There were no significant differences between study groups, no significant changes over time and no interactions between study groups and time of assessment in mean arterial pressure (**c**); cardiac output (**d**); systemic vascular resistance (**e**); and mixed venous oxygen saturation (**f**). *Polymyxin B-immobilized fiber hemoperfusion was carried out for 3 h

Figure 1 depicts dynamics of key hemodynamic parameters between study groups. There was a significant change over time—similar in both groups—in heart rate (*p* < 0.001), the vasopressor dependency index (*p* = 0.030), the inotropic score index (*p* = 0.028), pulmonary vascular resistance (*p* = 0.028), the Pv-aCO_2_ (*p* < 0.001) and the fluid balance (*p* = 0.048), whereas there were no effects associated with the use of PMX-HP, no significant change over time and no significant interaction between treatment in respect of the extraction ratio, the oxygen consumption and delivery. In all animals, after bacterial challenge, the median decrease in MAP, within 24 h, was 9.89 % (range 1.36–20.6 %) (Additional file 1: Figure S2). Post hoc analysis of only animals with the most significant hemodynamic impairment—characterized by a drop in MAP greater than 10 %—is depicted in Table 2. Still, in this specific population, comparisons between study groups did not show any substantial difference in hemodynamic, pulmonary and clinical variables.

**Table 2:** Hemodynamic, pulmonary and clinical variables in animals with a drop in mean arterial pressure ≥10 %, within 24 h from bacterial inoculum

	Group		*p* value
	Control (six animals)	Treatment (five animals)	
*Hemodynamic variables*			
Heart rate (beats/min)	66.7 ± 18.6	75.0 ± 22.0	*0.031*
Central venous pressure (mm Hg)	7.7 ± 3.8	7.9 ± 2.7	0.653
Mean arterial pressure (mm Hg)	77.6 ± 8.3	77.9 ± 11.5	0.814
Mean pulmonary arterial pressure (mm Hg)	20.8 ± 4.7	21.5 ± 4.7	0.388
Cardiac output (L/min)	3.3 ± 1.1	3.6 ± 1.2	0.245
Systemic vascular resistance (dyne/s/cm^5^)	1837.2 ± 593.5	1703.4 ± 567.9	0.252
Pulmonary vascular resistance (dyne/s/cm^5^)	299.9 ± 127.6	274.5 ± 113.9	0.159
Vasopressor dependency index	0.25 ± 0.10	0.30 ± 0.17	0.385
Mixed venous saturation (%)	61.9 ± 11.4	60.3 ± 0.10	0.164
Pv-aCO_2_	8.52 ± 3.38	8.78 ± 3.15	0.678
Fluid balance (mL)	−48.9 ± 445.2	−73.5 ± 501.9	0.842
*Pulmonary variables*			
PaO_2_/FiO_2_	353.8 ± 55.3	340.4 ± 81.8	0.792
PaCO_2_	40.3 ± 4.2	40.7 ± 4.5	0.598
Lung elastance (cm H_2_O/L)	27.7 ± 9.2	25.3 ± 8.2	0.477
Pulmonary shunt (%)	8.8 ± 4.2	9.2 ± 4.1	0.635
*Clinical variables*			
Temperature (°C)	38.3 ± 1.2	39.0 ± 1.1	*<0.001*
White blood cells (cells/µL)	17.9 ± 6.8	13.7 ± 5.0	*0.004*
Neutrophils (%)	45.6 ± 14.6	42.5 ± 12.9	0.364
Lymphocytes (%)	46.1 ± 14.8	50.4 ± 13.3	0.234
Monocytes (%)	2.5 ± 1.2	3.3 ± 2.8	0.631
Hb (g/dL)	8.6 ± 1.2	8.2 ± 1.5	0.290
Platelet (cells/mm^3^)	255.0 ± 61.8	258.2 ± 115.1	0.741
Creatinine (mg/dL)	0.99 ± 0.2	1.3 ± 1.4	0.154

Of note, only animals with a decrease in mean arterial pressure ≥10 %, within 24 h from bacterial inoculum, were included in this post hoc analysis

Italics *p* values indicate statistically significant differences between study groups

*Hb* hemoglobin, *PaCO*_*2*_ arterial partial pressure of carbon dioxide, *PaO*_*2*_ */FiO*_*2*_ ratio of partial pressure arterial oxygen and fraction of inspired oxygen, *Pv-aCO*_*2*_ venous-to-arterial partial pressure carbon dioxide difference

### Endotoxin clearance

**Fig. 2:**
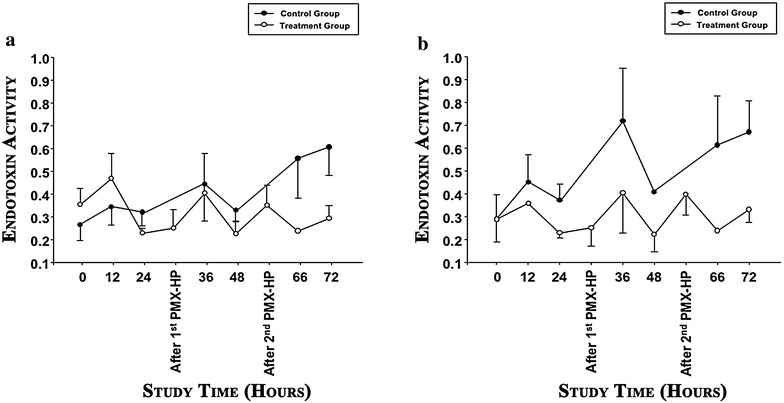
Endotoxin activity in the control and treatment group during study time. Data are reported as mean and standard error. Polymyxin B-immobilized fiber hemoperfusion was carried out for 3 h, after 30 and 50 h. **a** Endotoxin activity in all subjects. Group effect, *p* = 0.224; study time effect, *p* = 0.882; group * study time effect, *p* = 0.572. **b** Post hoc analysis in only animals with a decrease in mean arterial pressure ≥10 %, within 24 h from bacterial challenge. Group effect, *p* = 0.018; study time effect, *p* = 0.473; group * study time effect, *p* = 0.830

As depicted in Fig. 2a, PMX-HP, in comparison with standard treatment, had no effects in the endotoxin removal. Of note, blood EA raised recurrently in both groups, specifically after 36 h of MV, and at the end of the study. Nevertheless, subgroup analysis of only animals with the most significant hemodynamic impact (Fig. 2b) showed a significant reduction in blood EA from 0.50 ± 0.29 in the control group to 0.29 ± 0.14 in the PMX-HP group (*p* = 0.018).

### Clinical and pulmonary parameters

**Fig. 3:**
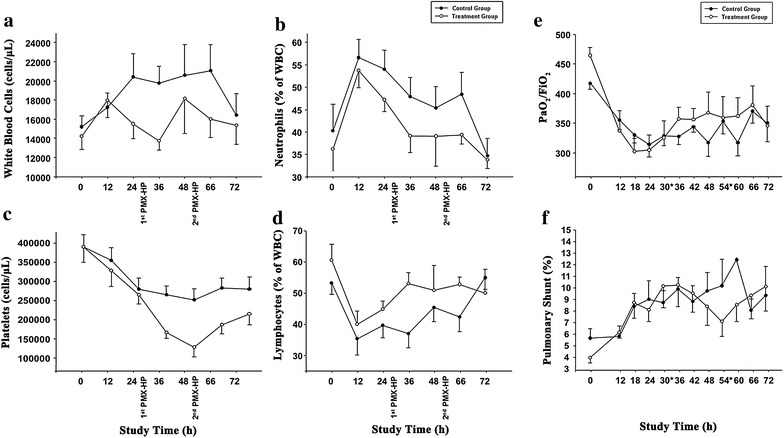
Differences in laboratory parameters and oxygenation parameters between the control and treatment group. **a** Total white blood cells count (**a**) (*p* = 0.031) differed between study groups, without significant changes over time, and no interactions between study groups and time of assessment; while percentage of neutrophils (**b**) differed between study groups (*p* = 0.009), significantly changed over time (*p* < 0.001), without interactions between study groups and time of assessment; similarly, percentage of lymphocytes (**c**) differed between study groups (*p* = 0.002), significantly changed over time (*p* < 0.001), without interactions between study groups and time of assessment; also, platelets count (**d**) differed between study groups (*p* < 0.001), significantly changed over time (*p* < 0.001), without interactions between study groups and time of assessment. Ratio of partial pressure arterial oxygen and fraction of inspired oxygen (PaO_2_/FiO_2_) (**e**) and pulmonary shunt (**f**) significantly changed over time (*p* < 0.001 and *p* = 0.018, respectively), in a similar fashion between groups, and without interactions between study groups and time of assessment. Of note, hemoperfusion through a column containing polymyxin B-immobilized fiber caused a significant reduction in circulating neutrophils and platelets and an increase in circulating lymphocytes

Figure 3 depicts clinical and pulmonary parameters throughout the study time. PMX-HP significantly decreased white blood cells (WBC) count and platelet counts. Furthermore, there was a significant change over time—similar in both groups—in body temperature (*p* < 0.001) and hemoglobin (*p* < 0.001), whereas there were no effects associated with the use of PMX-HP, no significant change over time and no significant interaction between treatment and time of assessment, in respect of prothrombin time, activated partial thromboplastin time and creatinine. Finally, alanine transaminase was 29.7 ± 9.8 and 34.6 ± 7.8 IU/L in the control and treatment group, respectively (*p* = 0.006), without significant changes over time, and no interactions between study groups and time of assessment. As for additional pulmonary parameters, arterial partial pressure of carbon dioxide significantly increased during the study time (*p* = 0.001), without differences between study groups, while there were no significant differences between study groups, no significant changes over time, and no interactions between study groups and time of assessment in lung elastance.

## Discussion

This study shows that in pigs with severe, untreated, *P. aeruginosa* pneumonia, PMX-HP decreases circulating neutrophils and only in animals with the most significant hemodynamic impairment after bacterial challenge, it significantly reduces blood endotoxin, but with no other benefits on hemodynamic and pulmonary parameters.

Up to the present time, PMX-HP has been primarily used in surgical patients with intra-abdominal sepsis. In 2009, the EUPHAS trial [8] demonstrated a survival benefit in patients with abdominal sepsis treated with surgery and PMX-HP. The study was designed to evaluate whether the use of PMX-HP could improve hemodynamic stability and decrease vasopressor requirements. At the first interim analysis, a significant survival benefit was found, and thus the study was terminated earlier. Yet, it is important to emphasize that the study was likely underpowered to show any reduction in mortality, which was obtained after adjustment for the sepsis-related organ failure assessment score. Also, the very high mortality rate in the control group limited the possibility to translate these findings into other intensive care unit populations. A later study by Puyen et al., in patient with peritonitis due to gut or biliary tract perforation, was specifically designed to detect differences in 28-day mortality, but a mortality rate of 27.7 and 19.5 % was found in the treatment and control group, respectively (*p* = 0.14). In an ongoing American trial (Evaluating the Use of Polymyxin B Hemoperfusion in a Randomized Controlled Trial of Adults Treated for Endotoxemia and Septic Shock (EUPHRATES), ClinicalTrials.Gov NCT01046669) [25], all patients with septic shock, irrespective of the source of infection, and EA ≥0.60 units are being treated with PMX-HP or standard treatment. Finally, in a recent Japanese retrospective propensity-matched analysis, postoperative polymyxin B hemoperfusion did not show any survival benefit in patients with abdominal septic shock [26]. Thus, it is important to emphasize that our laboratory study is the first that specifically assessed the effects of PMX-HP on severe *P. aeruginosa* pulmonary infection.

In our main analysis, PMX-HP did not decrease EA. Yet, post hoc analyses in a subset of animals with the worst decrease in MAP found a significant reduction in EA with the use of PMX-HP. Theoretically, these were the animals that could have benefited the most by endotoxin clearance, but this did not result in any valuable clinical improvement. Our results are in contrast to previous findings obtained from different animal models of septic shock [1]. The lack of severe cardiovascular dysfunction and tissue hypoperfusion found in our model, likely due to a compartmentalization of the infection, and swift hemodynamic support, could partially explain our negative findings. Indeed, as depicted in Fig. 1, after bacterial challenge, systemic vascular resistances decreased within the first 36 h, although not significantly, but there was a concomitant increase in central venous pressure, driven by rapid resuscitation with crystalloids and vasoactive drugs. Another potential reason is that in our model pulmonary pathogens constituted a continuous source of endotoxin. Conversely, in the majority of above-mentioned clinical studies, PMX-HP was applied following surgical control of the primary source of infection, and it was primarily aimed at clearing the residual circulating endotoxin. In our settings, we applied PMX-HP for 3 h, for two subsequent days. In an interesting recent trial [27], sequential estimation of PT-international normalized ratio (INR) was a strong predictor of the efficacy of treatment. Additionally, exacerbation of PT-INR (>0.16) before a second PMX-HP session was the most important predictor for 28-day mortality and futility of treatment. In our settings, we did not focus specifically on PT-INR. Yet, we can speculate that two sessions of PMX-HP may have not been sufficient to control the sustained surge of endotoxin from the lungs. Also, we might have started treatment relatively late, given that the first PMX-HP session started 24 h after the extensive bacterial challenge. Finally, given the most recent findings [28, 29] on pathogen-associated molecular patterns, endogenous alarmins and the host response through toll-like receptors, it could be argued that clearance of endotoxin could only have a limited impact on the complexity of severe respiratory Gram-negative infections.

In line with previous studies [11, 30, 31], we found a significant reduction of WBC, specifically neutrophils. During PMX-HP treatment, neutrophils bind with endotoxin, and then the endotoxin–neutrophil complex attaches to polymyxin [4]. Theoretically, this could provide some advantages during the course of sepsis, since the reduction in activated inflammatory cells could hinder the inflammatory cascade. Yet, polymorphonuclear leukocytes provide the front line of host defense and substantial removal could also facilitate the establishment or persistence of the infection. Furthermore, we found a significant decrease in platelets count, due to their absorption and consumption through the hemoperfusion column; nevertheless, this was not associated with any complication. Also, it should be highlighted that neither bleeding nor systemic and circuit clotting occurred with the use of PMX-HP.

Some investigators implied that PMX-HP could be useful in patients with severe pulmonary inflammations. During severe pulmonary bacterial infection, endotoxin induces a vast production of interleukin 8, which acts as a chemo-attractant. As a result, neutrophils migrate into the pulmonary interstitial space releasing inflammatory mediators. This drastically impairs the alveolar-capillary barrier and pulmonary function [32, 33]. In a previous study in rats, challenged intratracheally with endotoxin [34], PMX-HP reduced pulmonary chemotaxis and improved microcirculation, vascular permeability and oxygenation. In other clinical studies in patients with sepsis, mainly caused by pulmonary sources, investigators found a drastic decrease in the activation of chemokines, neutrophils and vascular endothelial cells with PMX-HP [13, 35], as a result pulmonary function improved. In the present study, we did not find any improvement in PaO_2_/FiO_2_ between groups. This was likely related to the lack of PMX-HP efficacy on endotoxin clearance. Additionally, it is important to consider that the pulmonary inflammatory cascade and neutrophils migration is a highly regulated mechanism that requires time to be modulated [13]; thus, even if we found a significant decrease in circulating neutrophils, we may have missed any improvement in pulmonary function, due to the limited duration of our study.

There are a few limitations to our study. First, we developed severe pneumonia in previously healthy and young animals. Thus, in comparison with the clinical scenario, we may have underestimated the pulmonary and hemodynamic response to pneumonia; additionally, we may have also undervalued the clinical benefit of PMX-HP in septic patients with comorbidities. Second, upon autopsy, we did not perform histology studies; thus, we may have missed significant additional benefits associated with clearance of pulmonary endotoxin. Third, as shown in Additional file 2, we found a mildly variable association between EA and *P. aeruginosa* or *E. coli* blood endotoxin concentration. Importantly, following formation of endotoxin–antibody complexes the assay utilizes polymorphonuclear leukocytes’ chemiluminescence to compute the EA [23]. Previous laboratory studies corroborated the applicability of the EA assay for the measurement of endotoxin in canine blood samples [36]. Yet, in pig’s blood, the oxidant release by neutrophils and the resulting light emission could vary in comparison with humans. Thus, the absolute values of endotoxin concentration and EA of our study are useful, but must be interpreted with caution. Additionally, it should be emphasized that some investigators recently questioned the reliability of EA assay in humans versus older methods, such as the limulus amebocyte lysate assay [37]. Fourth, during acute stresses, release of quiescent endotoxin into the bloodstream by endogenous Gram-negative flora of the gastrointestinal tract is substantial in humans [38–41]. This may not be relevant in pigs with pneumonia and could have further underestimated the effects of PMX-HP. Fifth, in the present study, and in previous similar studies in septic pigs [42], the infection was untreated. This was aimed at studying the effects of PMX-HP in a scenario of sustained infection and consistent high level of endotoxemia. Yet, in clinical settings, antibiotic-induced release of endotoxin from pathogens could be significant [43, 44], particularly during the first hours of antimicrobial treatment. Consistently, in previous positive clinical study [8], patients were treated with antibiotics—following abdominal surgery—and PMX-HP helped clearing the residual endotoxin. Thus, we cannot fully reject the synergistic effects of PMX-HP in treated patients, which should be further evaluated in future investigations. Finally, our study lacks of a control group without pulmonary bacterial challenge. This additional group could have further increased the scientific value of the study and comparisons among groups.

## Conclusions

In conclusion, we showed that in pigs with severe untreated *P. aeruginosa* pneumonia, PMX-HP does not improve the early management of the severe infection. Confirmation of these important negative findings in different animal species and in humans is warranted, to corroborate the no benefits associated with the use of PMX-HP in severe Gram-negative pulmonary infections.

## Additional files

10.1186/s13613-016-0155-3 Supplementary figures.
10.1186/s13613-016-0155-3 Appendix: Preliminary studies to evaluate the applicability of the Endotoxin Activity Assay^TM^ to measure endotoxin activity in pig whole blood.

## Abbreviations

ALI: acute lung injury
CO: cardiac output
CVP: central venous pressure
cfu: colony-forming unit
EA: endotoxin activity
PMX-HP: hemoperfusion through the column containing polymyxin B-immobilized fiber
MV: mechanical ventilation
PAP: pulmonary arterial pressure
PCWP: pulmonary artery wedge pressure
WBC: white blood cells
MAP: mean arterial pressure
SvO_2_: mixed venous oxygen saturation
PTT: partial thromboplastin time
PVR: pulmonary vascular resistance
SV: stroke volume
SVR: systemic vascular resistance
Pv-aCO_2_: venous-to-arterial partial pressure carbon dioxide difference
