# Supplementary material for: The effects of direct hemoperfusion using a polymyxin B-immobilized column in a pig model of severe Pseudomonas aeruginosa pneumonia
Source: Ann Intensive Care. 2016 Jul 5;6:58. doi: 10.1186/s13613-016-0155-3 (PMC4932027; doi:10.1186/s13613-016-0155-3)

**SUPPLEMENTARY FIGURES**

**THE EFFECTS OF Direct Hemoperfusion Using a Polymyxin B Immobilized Column in a pig model of severe *Pseudomonas aeruginosa* pneumonia**

Gianluigi Li Bassi^1,2,3^; Joan Daniel Marti^1,2,3^; Eli Aguilera Xiol^1,3^; Talitha Comaru^1,2^; Francesca De Rosa^1,4^; Montserrat Rigol^1,2^; Silvia Terraneo^1,4^; Mariano Rinaudo^1^; Laia Fernandez^1,2,3^; Miguel Ferrer^1,2,3^ and Antoni Torres^1,2,3,5^.

**FIGURE LEGENDS:**

**Figure S1**

After 24 and 48 hours from bacterial challenge, animals were placed in the supine position and hemoperfusion through a column containing polymyxin B-immobilized fiber was performed for 3 hours, through a veno-venous, pump-driven, extracorporeal system. A dual lumen catheter was inserted into the femoral vein for blood drainage and return. A, Blood Drainage; B, Blood Return; C, Extracorporeal System; D, Mechanical Pump; E, Column containing polymyxin B-immobilized fiber; F, Heparin pump.

**Figure S2**

Frequency distribution (percentage of observations) of the decrease in mean arterial pressure vs. baseline values, within the first 24 hours from bacterial challenge. Mean ± standard deviation and median values were 9.81±4.33% and 9.89%, respectively. The decrease in mean arterial pressure vs. baseline values ranged from 1.36% to 20.6%.

Figure S1


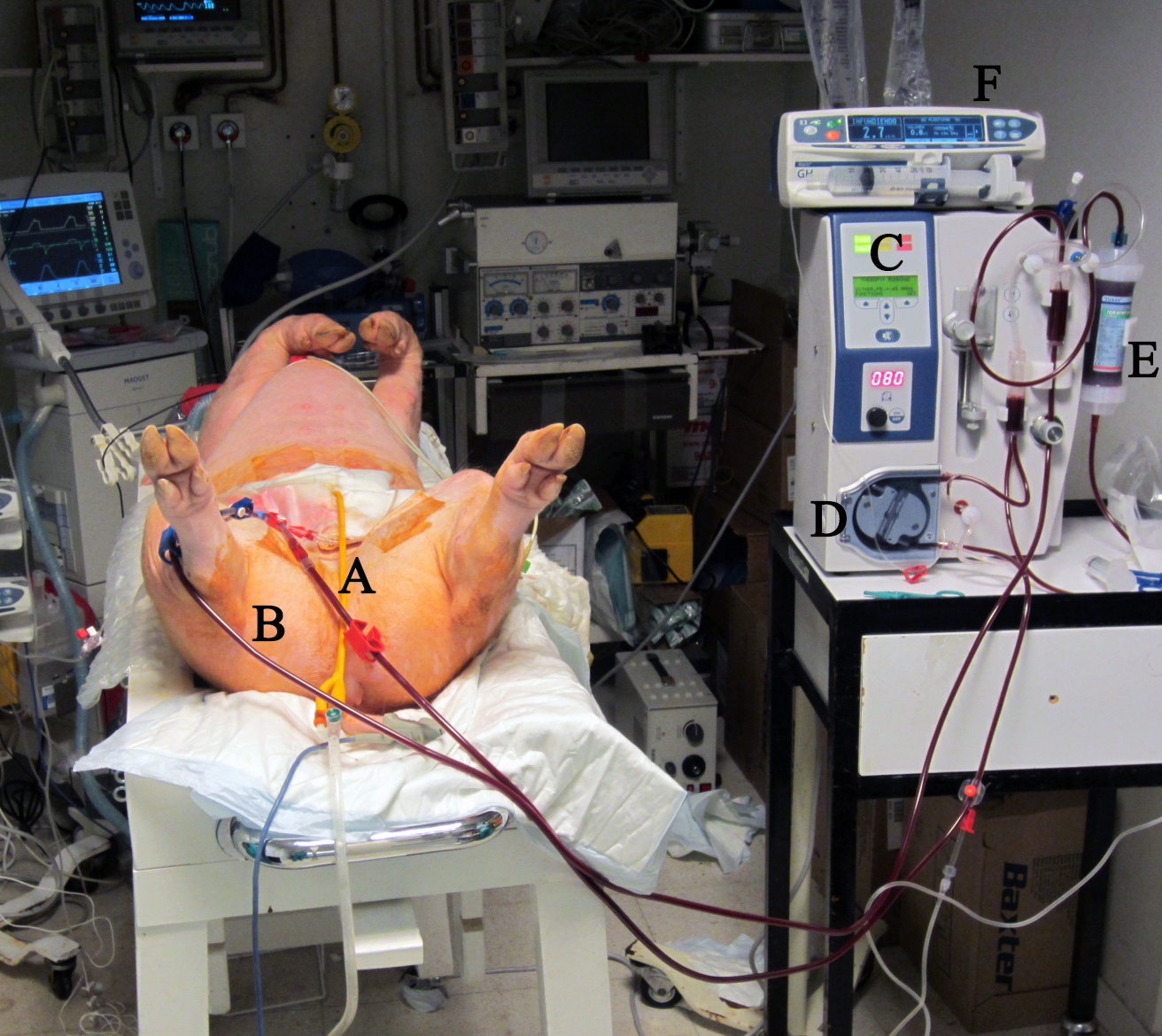


Figure S2


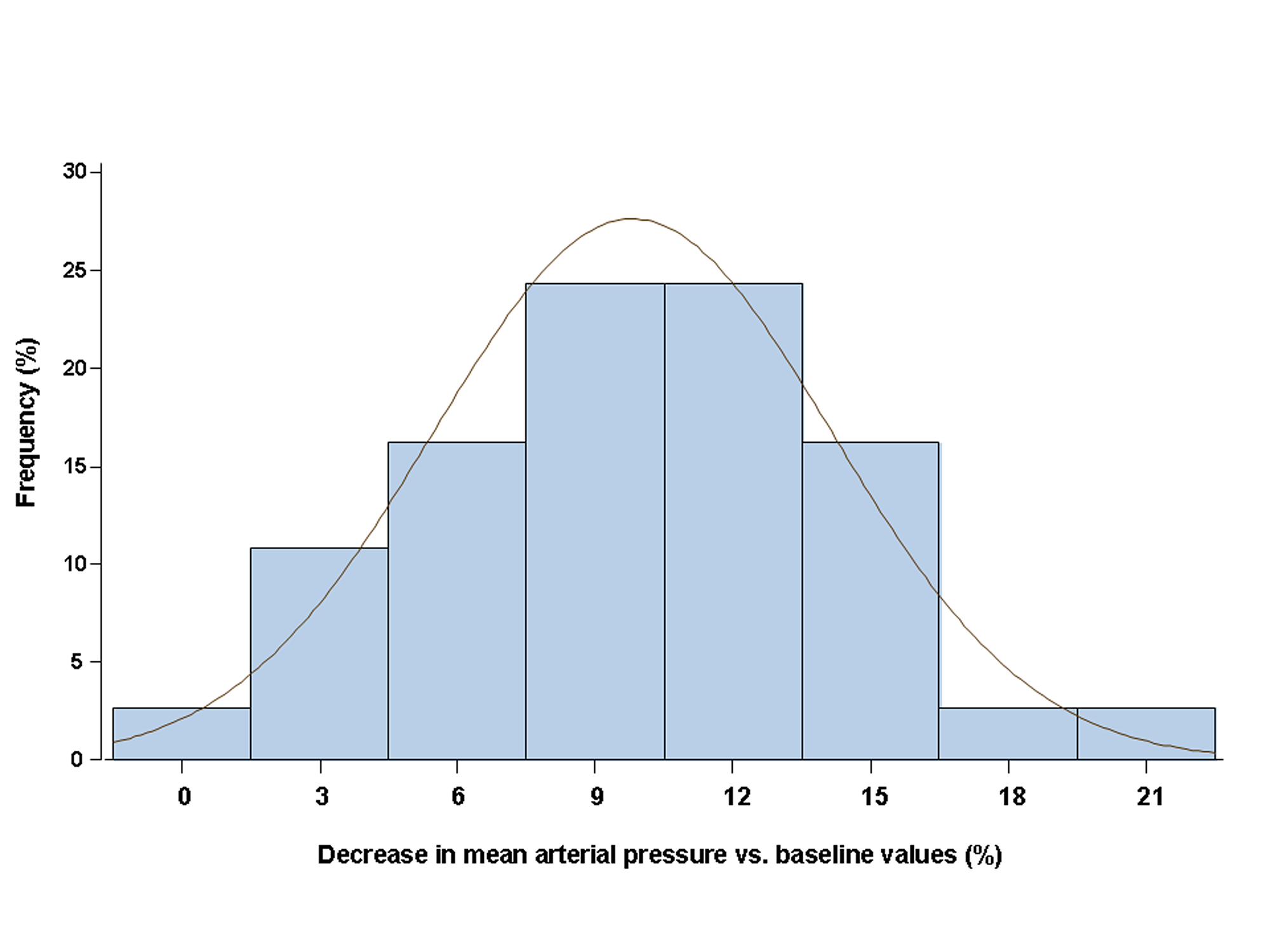

Supplement: Supplementary file 1 — 10.1186/s13613-016-0155-3 Supplementary figures. [file 13613_2016_155_MOESM1_ESM.docx]
