# Supplementary material for: The effects of direct hemoperfusion using a polymyxin B-immobilized column in a pig model of severe Pseudomonas aeruginosa pneumonia
Source: Ann Intensive Care. 2016 Jul 5;6:58. doi: 10.1186/s13613-016-0155-3 (PMC4932027; doi:10.1186/s13613-016-0155-3)

**APPENDIX**

**Preliminary studies to evaluate the applicability of the ENDOTOXIN ACTIVITY ASSAY^TM^ to measure endotoxin activity in pig whole blood**

**Materials and Methods**

We performed preliminary analyses using pig’s blood, obtained from 4 healthy Large-White Landrace pigs, and challenged with *Escherichia Coli* and *Pseudomonas Aeruginosa* endotoxin to validate Endotoxin Activity Essay (EAA).

Two 7.5 mL pig blood samples were collected into EDTA test tubes. We prepared 100-fold concentrated endotoxin solutions for both *E. coli* and *P. aeruginosa* endotoxin, using fourteen 1.5-mL sterile eppendorf tubes as follows:

- 1. We instilled 10 µL of endotoxin (1mg/mL) into 990 µL of NaCL 0.9% solution (resulting endotoxin concentration: 10 µg/mL)
  2. We instilled 32 µL of above mentioned 10 µg/mL endotoxin solution into 968 µL of NaCl 0.9% (resulting endotoxin concentration: 3200 pg/mL)
  3. We instilled 100 µL of above mentioned 3200 pg/mL endotoxin solution into 300 µL of NaCl 0.9% (resulting endotoxin concentration: 800 pg/mL)
  4. We instilled 100 µL of above mentioned 800 pg/mL endotoxin solution into 300 µL of NaCl 0.9% (resulting endotoxin concentration: 200 pg/mL)
  5. We instilled 100 µL of above mentioned 200 pg/mL endotoxin solution into 300 µL of NaCl 0.9% (resulting endotoxin concentration: 50 pg/mL)
  6. We instilled 100 µL of above mentioned 50 pg/mL endotoxin solution into 300 µL of NaCl 0.9% (resulting endotoxin concentration: 12.5 pg/mL)
  7. Finally, we prepared 400 µL of NaCl 0.9% solution (resulting endotoxin concentration: 0 pg/mL)

We transferred 12 uL from each vial (endotoxin concentration range: 3200-0 pg/mL) into 12 sterile 1.2 mL empty tubes. We added 1.2 mL of pig’s blood into each of the twelve 100-fold concentrated endotoxin solutions and vortexed the sample. Then, the samples were maintained at room temperature for 10 min. Finally, we evaluated Endotoxin Activity Assay for each tube (12 tests).

*Statistical analysis*

Linear and non linear regression analyses were applied to assess the association between EA and *P.aeruginosa* or E.coli endotoxemia and to find the best predictive model. A two-sided p value < 0.05 was considered statistically significant. All analyses were performed using SAS 9.2 software.

**Results**

In the figure below is reported the Endotoxin Activity Assay of pig blood challenged with *P.aeruginosa*, and *E.coli* endotoxin. *E.coli* endotoxin showed the strongest correlation with the EAA.

**Appendix Figure Legend**

**Appendix Figure 1**

A, Exponential regression analysis of the association between *Pseudomonas Aeruginosa* Endotoxin Concentration and Endotoxin Activity Assay, N:15. B, Linear regression analysis of the association between *Escherichia Coli* Endotoxin Concentration and Endotoxin Activity Assay, N:14. C, Sigmoidal regression analysis of the association between *Pseudomonas Aeruginosa* and *Escherichia Coli* Endotoxin Concentration and Endotoxin Activity Assay. N:29.

**Supplementary on-line Appendix Figure 1**
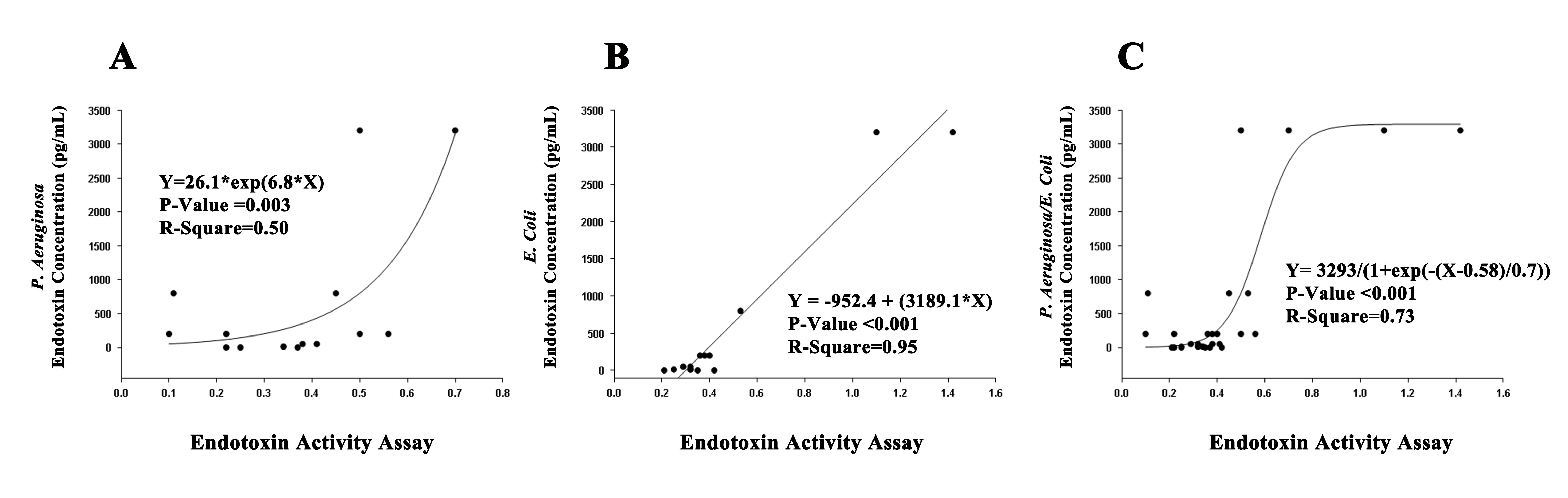

Supplement: Supplementary file 2 — 10.1186/s13613-016-0155-3 Appendix: Preliminary studies to evaluate the applicability of the Endotoxin Activity AssayTM to measure endotoxin activity in pig whole blood. [file 13613_2016_155_MOESM2_ESM.docx]
